# Mechanistic insights into immune checkpoint inhibitor-related hypophysitis: a form of paraneoplastic syndrome

**DOI:** 10.1007/s00262-021-02955-y

**Published:** 2021-05-11

**Authors:** Keitaro Kanie, Genzo Iguchi, Hironori Bando, Shin Urai, Hiroki Shichi, Yasunori Fujita, Ryusaku Matsumoto, Kentaro Suda, Masaaki Yamamoto, Hidenori Fukuoka, Wataru Ogawa, Yutaka Takahashi

**Affiliations:** 1 Division of Diabetes and Endocrinology, Department of Internal Medicine, Kobe University Graduate School of Medicine, Kobe, Japan; 2 Division of Diabetes and Endocrinology, Kobe University Hospital, Kobe, Japan; 3 Medical Center for Student Health, Kobe University, Kobe, Japan; 4 Department of Diabetes and Endocrinology, Nara Medical University, Nara, 840 Shijou-cho, Kashiihara, Nara, 634-8521 Japan

**Keywords:** Autoimmunity, Immune checkpoint inhibitor, Hypopituitarism, Hypophysitis, Paraneoplastic syndrome

## Abstract

**Background:**

Immune checkpoint inhibitors (ICIs) as a cancer immunotherapy have emerged as a treatment for multiple advanced cancer types. Because of enhanced immune responses, immune-related adverse events (irAEs), including endocrinopathies such as hypophysitis, have been associated with the use of ICIs. Most underlying mechanisms of ICI-related hypophysitis remain unclear, especially for programmed cell death-1 (PD-1)/PD-1 ligand 1 (PD-L1) inhibitors. We hypothesized that ICI-related hypophysitis is associated with paraneoplastic syndrome caused by ectopic expression of pituitary-specific antigens.

**Methods:**

Twenty consecutive patients with ICI-related hypophysitis between 2017 and 2019 at Kobe University Hospital were retrospectively analyzed. Circulating anti-pituitary antibodies were detected using immunofluorescence staining and immunoblotting. Ectopic expression of pituitary autoantigens in tumor specimens was also examined.

**Results:**

Eighteen patients were treated with PD-1/PD-L1 inhibitors, and two were treated with a combination of cytotoxic T-lymphocyte antigen-4 (CTLA-4) and PD-1 inhibitors. All patients showed adrenocorticotropic hormone (ACTH) deficiency and additionally, three showed thyroid-stimulating hormone (TSH) deficiency, and one showed gonadotropin-releasing hormone (GnRH) deficiency. Among these patients, three exhibited anti-pituitary antibodies, two with anti-corticotroph antibody and one with anti-somatotroph antibody. Interestingly, the anti-corticotroph antibody recognized proopiomelanocortin (POMC) and those two patients exhibited ectopic ACTH expression in the tumor, while the patients without anti-corticotroph antibody did not.

**Conclusions:**

We demonstrated 10% of PD-1/PD-L1 inhibitors-related hypophysitis were associated with the autoimmunity against corticotrophs and maybe caused as a form of paraneoplastic syndrome, in which ectopic expression of ACTH in the tumor was observed. It is also suggested that the pathophysiology is heterogenous in ICI-related hypophysitis.

**Supplementary Information:**

The online version contains supplementary material available at 10.1007/s00262-021-02955-y.

## Introduction

The discovery of immune checkpoint inhibitors (ICIs) has revolutionized cancer treatment and has been shown to be effective for several types of advanced cancer [1]. However, these agents are associated with significant potential toxicities termed immune-related adverse events (irAEs). Particularly, several endocrinopathies, including hypophysitis, are often observed with the use of these agents [2]. However, the underlying mechanisms of these irAEs remain largely unknown.

Cytotoxic T-lymphocyte antigen-4 (CTLA-4) expressed on T cells suppresses T-cell activation, and the inhibition of CTLA-4 leads to T-cell activation and the inhibition of regulatory T cells [3, 4]. Interestingly, CTLA-4 is also expressed in the pituitary gland and may be directly involved in the development of hypophysitis [5]. On the other hands, programmed cell death-1 (PD-1) is mainly expressed on effector T cells [6] and binds to programmed cell death-1 ligand 1 (PD-L1) expressed by tumor cells.

Secretion of thyroid-stimulating hormone (TSH) and luteinizing hormone (LH)/follicle-stimulating hormone (FSH) is frequently impaired in the hypophysitis associated with CTLA-4 inhibitor therapy, along with impairment in adrenocorticotropic hormone (ACTH) secretion [7]. In contrast, PD-1 inhibitor also induces hypophysitis, but less frequently [8], with most of these patients developing isolated ACTH deficiency (IAD) [9]. Patients with PD-L1 inhibitor-related hypophysitis also develop IAD [10]. In addition, at the diagnosis of sellar masses in patients treated with ICIs, it should be taken into account that not PD-1/PD-L1-related hypophysitis but CTLA-4 inhibitor-related hypophysitis often reveals pituitary enlargement with headache; however, it is important to exclude a metastasis of the malignancy [11]. These data strongly suggest that the underlying mechanisms of PD-1 or PD-L1 inhibitor-related hypophysitis are different from those in CTLA-4 inhibitor therapy.

The importance of anti-pituitary antibodies (APAs) in pituitary autoimmunity associated with hypophysitis has been widely recognized [12]. In fact, several kinds of pituitary autoantibodies against thyrotrophs, corticotrophs, and gonadotrophs have been reported in patients with ICI-related hypophysitis [5]; however, it is currently unknown whether these autoantibodies play a causal role. Anti-corticotroph antibody has also been detected in patients with IAD, in which autoimmunity has been considered to be involved [13, 14]. One patient with IAD exhibited circulating anti-corticotroph antibody, as well as cytotoxic T cells that specifically recognize proopiomelanocortin (POMC) [15]. Interestingly, this patient’s complicated with a tumor that ectopically expressed POMC, suggesting that the IAD was caused by a form of paraneoplastic syndrome in the case.

In the current study, we hypothesized that ICI-related hypophysitis was caused as a paraneoplastic syndrome and aimed to clarify the significance of APAs.

### Materials and methods

#### Patients

This study was approved by the ethics committee of Kobe University Graduate School of Medicine (#29–62). All methods were performed in accordance with the guidelines of the approved protocol. Patients provided written informed consent. Most patients were treated in Hyogo Cancer Center, and the diagnosis of ICI-related hypophysitis was performed in Kobe University Hospital. Twenty consecutive patients who diagnosed with ICI-related hypophysitis were enrolled. Most patients were treated with PD-1/PD-L1 inhibitors rather than CTLA-4 inhibitors because of the historical background in Japan.

#### Diagnosis of ICI-related hypophysitis and hormone assays

For the screening of hypopituitarism, basal levels of pituitary and peripheral hormones were measured [16]. In patients with a suspicion of hypopituitarism, provocation tests for anterior pituitary hormones and pituitary MRI were performed [17]. Provocative test was performed as previously described [17, 18] using insulin (0.05 unit/kg) or corticotropin-releasing hormone (CRH) (100 µg), thyroid-releasing hormone (TRH) (200 µg), luteinizing hormone-releasing hormone (LHRH) (100 µg), and growth hormone-releasing peptide-2 (GHRP-2) (100 µg). ICI-related hypopituitarism was defined and diagnosed according to the guidelines for endocrine-irAEs [16, 19, 20]. If basal level of serum cortisol was less than 4 µg/dL and there was no increase in basal ACTH level, we diagnosed ACTH deficiency. Growth hormone (GH), prolactin (PRL), TSH, LH, and FSH deficiencies were diagnosed by baseline hormone levels of the pituitary and its peripheral hormones. We diagnosed ACTH deficiency by decreased peak serum cortisol value (< 18 µg/dL) and impaired responses of ACTH (< twofold of baseline) in CRH test and TSH deficiency by impaired responses of TSH (< 10 µIU/mL) in TRH test. No patients had a history of exogenous steroid administration. Patients who underwent radiotherapy to the hypothalamus and/or pituitary area and have a history of pituitary surgery were excluded. Case 2 received Cyberknife radiosurgery for metastatic lesions in the brain; however, the hypothalamus and pituitary area were not exposed to radiation. The plasma ACTH, cortisol, and GH levels were measured by a chemiluminescent enzyme immunoassay (CLEIA; TOSOH, Tokyo, Japan), and PRL, TSH, free thyroxine 4 (T4), LH, FSH, estradiol (E2), and, testosterone levels were measured by a chemiluminescent immunoassay (CLIA; Abbott, Tokyo, Japan), and IGF-I level was assayed by an electrochemiluminescence immunoassay (ECLIA; Roche, Tokyo, Japan), respectively.

#### Animals

Experiments with animal tissues were performed according to the guidelines of the Animal Ethics Committee of Kobe University Graduate School of Medicine. The experimental protocols were approved by the Institutional Animal Care and Use Committee. Mouse pituitary tissues were used for immunohistochemical analyses.

#### Immunofluorescence staining and immunohistochemistry

For immunofluorescence staining of mouse pituitary specimens, tissues were fixed by perfusion with 4% paraformaldehyde, underwent heat-induced antigen retrieval with tris-ethylenediaminetetraacetic acid (EDTA) buffer (10 mM Tris base, 1 mM EDTA solution, 0.1% Tween 20, pH 9.0), and were permeabilized for 15 min at room temperature using phosphate-buffered saline (PBS) supplemented with 0.3% Triton X-100. The specimens were blocked using Blocking One buffer (Cat# 05,999–84, Nacalai Tesque, Kyoto, Japan), Fc Receptor Blocker (Cat# NB335, Innovex Biosciences, Richmond, CA, the USA), and True-Black Quencher (Cat# 23,007, Biotium, Hayward, CA, the USA). Subsequently, specimens were incubated with patient sera (1:20) for 24 h at 4 °C, washed three times with PBS supplemented with 0.05% Tween-20 (PBS-T), and then incubated with goat anti-human-IgG-Alexa Flour 488 antibody (Cat# ab70328, Abcam) for 2 h at room temperature (RT). This was followed by 24 h incubation with pituitary hormone antibodies, including anti-ACTH (Cat# Ab74976, Abcam, Cambridge, MA, the USA), anti-GH (Cat# sc-166696, Santa Cruz), anti-PRL (Cat# A0569, Dako, Carpinteria, CA), anti-TSH (Cat# M3503, Dako), anti-LH (Cat# M350201-2, Dako), anti-FSH (Cat# M3504, Dako) and anti-S100β (Cat# Ab52642, Abcam) antibodies. The secondary antibodies were donkey anti-mouse Alexa Flour 546 (Cat# ab10036, Abcam) and donkey anti-rabbit Alexa Flour 546 (Cat# ab10040, Abcam) as appropriate and were used. The nuclei were counterstained with Hoechst 33,342.

For the POMC absorption test, each serum sample (1:20) was incubated 24 h at 4 °C with or without 150 μg/mL of recombinant human POMC (rhPOMC) protein (Cat# GWB-P0950A, GenWay Biotech) prior to use for immunofluorescence staining.

In the immunohistochemistry for the detection of ectopic hormone expression, at least three slices that covered most of tissues were analyzed because the ectopic expression was sometimes confined in a part of the tumor. The fixed tissues underwent antigen retrieval, were permeabilized, and blocked above-mentioned. Subsequently, specimens were incubated with primary antibodies anti-ACTH (Cat# M3501, Dako) and anti-GH (Cat# A0570, Dako). Color development was performed using 3,3'-diaminobenzidine (DAB) as a chromogen. We obtained all images using a BZ-X710 fluorescence microscope (Keyence, Osaka, Japan) and then reconstructed the images using BZ-H3A software (Keyence). The representative results from a part of patients without anti-POMC antibody are shown.

For autoantibody detection, 0.1 µg of rhPOMC (GenWay Biotech) was applied for SDS-PAGE and immunoblotted with the serum from each patient or with six healthy control sera as primary antibody (1:50). Anti-POMC antibody (Cat# SAB1410992; SIGMA) was used as a control. The representative results are shown.

## Results

### Clinical characteristics of patients with ICI-related hypophysitis

Twenty consecutive patients who diagnosed with ICI-related hypophysitis [20] were enrolled in the study. Sixteen patients were treated with PD-1 inhibitor, two were treated with PD-L1 inhibitor, and two were treated with a combination of CTLA-4 and PD-1 inhibitors (Table 1). The average duration of treatment before the onset of hypophysitis was 9.0 wk (range, 8.5–9.5 wk) for the combination therapy, 20.5 wk (range, 12.3–36.3 wk) for the PD-1 inhibitor therapy, and 54.0 wk (range, 53.0–55.0 wk) for the PD-L1 inhibitor therapy. With respect to anterior pituitary hormone impairment at the time of the diagnosis of the hypophysitis, all patients exhibited ACTH deficiency, and three additionally exhibited TSH deficiency and one LH/FSH deficiency, in which the ACTH deficiency in all patients was comparable with the previous report [8] (Table 1). In the six patients being treated with levothyroxine, the treatment had been started before the diagnosis of hypophysitis (Table 1). Pituitary MRI was performed in 17 patients. A slight enlargement was observed in three patients, slight atrophy in three patients, and no obvious abnormalities in 11 patients.

**Table 1:** Clinical characteristics of the patients

Age	Cease No	Sex	ICI	Primary disease	Duration of ICI administration before onset (weeks)	Pituitary Hormone deficiency	Anti-pituitary antibody	Previous endocrine irAE	ACTH (pg/mL)
76	1	M	PD-1 + CTLA4	Non-small cell lung cancer	8	ACTH	−		2.3
76	2	M	PD-1	Malignant melanoma	20	ACTH + TSH	Corticotroph		3.6
65	3	M	PD-L1	Non-small cell lung cancer	56	ACTH	−		4.1
71	4	M	PD-L1	Non-small cell lung cancer	52	ACTH	−		4.8
87	5	M	PD-1	Renal cell carcinoma	7	ACTH	−	Primary hypothyroidism	4.3
68	6	M	PD-1	Renal cell carcinoma	12	ACTH	−	Primary hypothyroidism	2.7
64	7	F	PD-1	Non-small cell lung cancer	23	ACTH	−		12.2
73	8	M	PD-1	Malignant melanoma	12	ACTH	−		7.5
65	9	F	PD-1	Stomach cancer	21	ACTH	−	Thyrotoxicosis	< 0.2
70	10	F	PD-1	Renal cell carcinoma	25	ACTH	−		4.5
53	11	M	PD-1	Stomach cancer	17	ACTH + TSH	Somatotroph		10.7
60	12	M	PD-1	Stomach cancer	48	ACTH + TSH	−		< 0.2
71	13	M	PD-1	Renal cell carcinoma	4	ACTH	−	Primary hypothyroidism	7.3
64	14	M	PD-1	Renal cell carcinoma	28	ACTH	Corticotroph		9.3
70	15	M	PD-1	Urinary tract cancer	12	ACTH	−		3.8
75	16	M	PD-1 + CTLA4	Esophageal cancer	10	ACTH	−		4.9
68	17	M	PD-1	Submandibular gland cancer	47	ACTH	−		10.6
35	18	F	PD-1	Large cell neuroendocrine carcinoma	29	ACTH + LH + FSH	−	Primary hypothyroidism	< 2
55	19	M	PD-1	Renal cell carcinoma	9	ACTH	−	Primary hypothyroidism	3.2
72	20	M	PD-1	Non-small cell lung cancer	28	ACTH	−	Primary hypothyroidism	3.6

Cortisol (µg/dL)	GH (ng/mL)	IGF-I (ng/mL)	TSH (µIU/mL)	FT4 (ng/dL)	PRL (ng/mL)	LH (µIU/mL)	FSH (µIU/mL)	T (pg/mL)	E2 (pg/mL)
0.3	0.4	95	2.2	1.2	16.1	6.5	12.8	N/A	N/A
0.8	0.2	78	4.1	0.7	13.6	4.7	6.9	2.7	N/A
< 0.2	0.4	47	1.5	0.8	17.8	4.4	9.4	6.4	N/A
3.1	0.1	61	1.3	1.1	11.1	24.3	50.4	5	N/A
1.9	4.1	79	2.0	1.08*	12.2	22.3	35.3	6.6	N/A
0.7	N/A	157	1.3	1.0*	25.6	25.6	49.6	3.4	N/A
0.8	0.2	40	4.3	0.9	17.1	10.6	24.1	N/A	< 10
0.7	0.5	91	4.9	1.0	13.2	2.8	7	6.7	N/A
2.4	2.4	49	0.05	2.5	21.1	36.6	95.7	N/A	< 10
7.2	0.4	69	4.5	1.0	132	34.9	49.5	N/A	< 10
2.5	1.3	92	0.4	0.8*	4.1	2.5	23.3	2.8	N/A
0.9	N/A	56	2.3	1.1	21.1	6.6	11.8	5.2	N/A
7.8	0.1	110	1.8	1.0*	10.1	7.9	20	7.8	N/A
2.9	0.6	46	3.2	1.1	14.5	9	21.2	7.7	N/A
0.9	0.1	164	2.4	0.8	17.5	8.3	16.1	7.4	N/A
0.6	0.3	104	9.9	0.7	14.4	10.5	22.2	5.5	N/A
1.8	0.2	109	1.4	1.1	10.4	2.6	3.9	8.2	N/A
3.1	0.6	113	1.9	1.1*	13.4	0.1	1.4	N/A	< 10
1.8	N/A	100	3.9	1.0*	11.1	4.6	15.5	5.6	N/A
7.7	N/A	63	18.8	1.0	13.2	6	12.9	0.1	N/A

### Anti-pituitary antibodies in patients with ICI-related hypophysitis

**Fig. 1:**
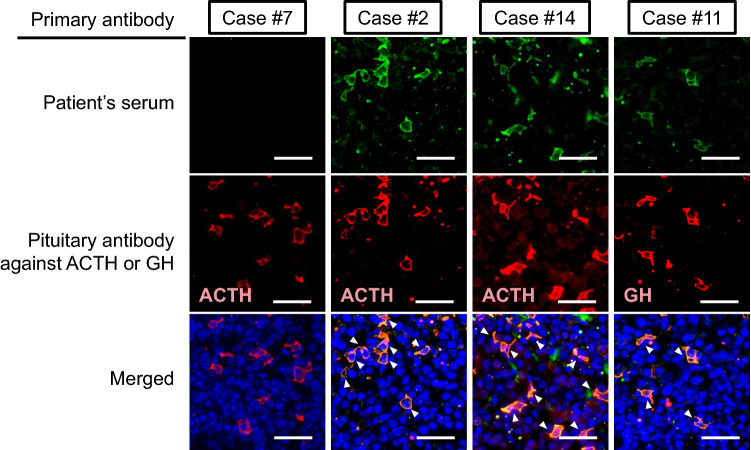
Immunofluorescence staining using patients’ serum and antibodies against pituitary hormones. Mouse pituitary tissue was stained with patients’ serum and anti-pituitary hormone antibodies. Serum of cases #2 and #14 recognized corticotrophs and that of case #11 recognized somatotrophs. Case #7 showed negative for circulating anti-pituitary antibody. Autoantibodies against other pituitary hormones were not detected (Supplementary Fig. 1). The representative results are shown. Scale bars; 100 µm

We evaluated the presence of circulating anti-pituitary antibodies using mouse pituitary tissue by immunofluorescent staining. Among the 20 patients, we detected anti-pituitary antibodies in three patients (15%) (Fig. 1 upper panel, Table 1). Next, we performed double staining analysis for each pituitary hormone (ACTH, TSH, GH, PRL, LH, and FSH) and S100β, demonstrating that the sera of two patients recognized corticotrophs and one recognized somatotrophs, while no merged signals were detected in the double staining with other pituitary hormone antibodies (Fig. 1 middle and lower panel, Supplementary File 1).

### Autoimmunity against POMC in the patients with anti-corticotroph antibody

**Fig. 2:**
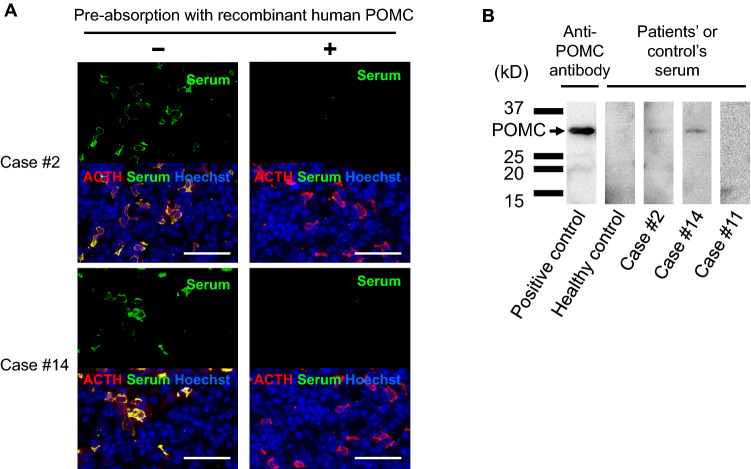
Antigen-absorption experiments using rhPOMC protein in immunofluorescence and immunoblotting analysis using patients’ serum. **a** Pre-absorption of the serum with rhPOMC protein diminished the signal in cases #2 and #14, indicating that the autoantibody specifically recognized POMC protein. Scale bar: 50 µm. **b** The sera in the cases #2 and #14 specifically recognized a 29-kDa protein that corresponded to POMC protein (arrow). The representative results are shown. No sera from other cases nor healthy subjects exhibited anti-POMC antibody

To clarify the corticotroph autoantigen in these patients, we performed a pre-absorption test using recombinant human POMC protein. Interestingly, pre-absorption of the sera with POMC protein diminished the reactivity against corticotrophs (Fig. 2a). In addition, immunoblotting using the patient sera (case #2 and #14) clearly demonstrated recognition of the POMC protein (Fig. 2b), indicating that the autoantibody was specific for POMC. Sera from the other patients and from healthy subjects did not show any signals (Fig. 2b, data not shown), indicating the specificity of this autoantibody.

### ACTH was ectopically expressed in tumor tissues of the patients with anti-POMC antibody

**Fig. 3:**
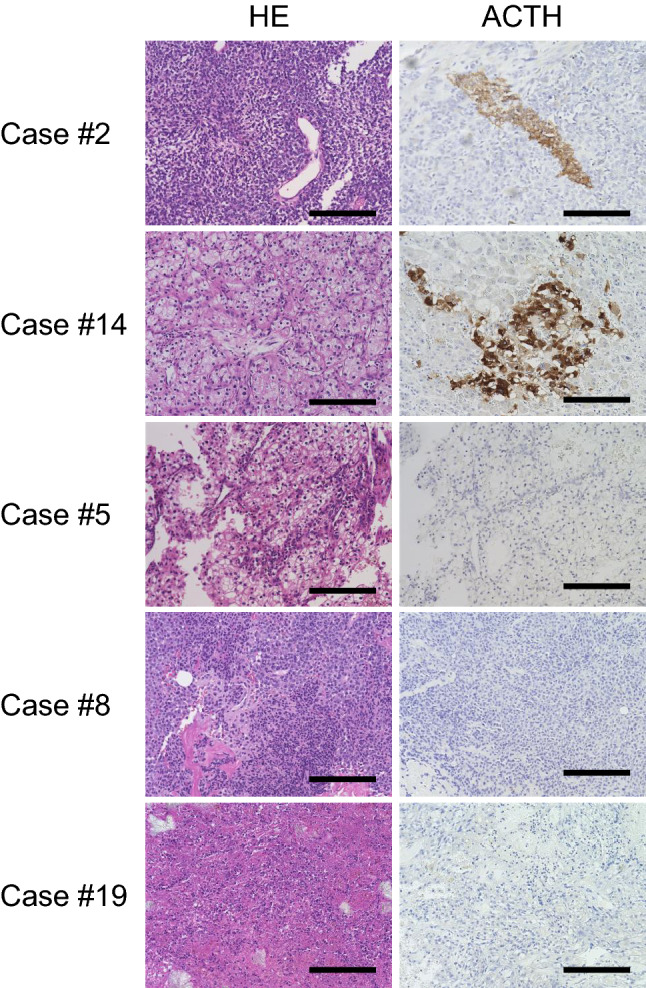
Specific ectopic ACTH expression was detected in the tumor specimens, in which case revealed anti-POMC antibody. Staining and immunohistochemistry for ACTH in the tumor tissues. Tumor specimen in the cases #2 and #14 specifically demonstrated ectopic ACTH expression. In contrast, tumor specimen in other cases was negative for ACTH. The representative results from a part of patients without anti-POMC antibody are shown. Scale bars: 200 µm

Because it has been reported that ectopic ACTH expression is not rare in various cancers [14, 15], we hypothesized that the tumor ectopically expressed ACTH, resulting in the evocation of autoimmunity against ACTH. Interestingly, the neoplastic cells of malignant melanoma in patient #2 and renal cell carcinoma in patient #14 specifically exhibited ectopic ACTH expression, while the other tumors of patients without anti-corticotroph antibody did not (Fig. 3). In contrast, ectopic GH expression was not detected in the patient with anti-GH antibody (data not shown).

## Discussion

Currently, the precise mechanisms of irAEs remain unclear; however, several biomarkers associated with the mechanisms have been suggested, including immune cell phenotypes [20], genetic variability of immune systems [21], and autoantibodies [5, 21]*.* Interestingly, in patients with metastatic melanoma treated with ipilimumab who developed irAEs, the repertoire of autoantibodies against self and cancer antigens preceded the development of irAEs [22]. In addition, increased T-cell activity against antigens present in tumors and normal tissues has been proposed as one of the underlying mechanisms [23]. These reports strengthened the importance of immune cross-reactivity between the tumor and involved organs as the mechanisms for irAE.

In the current study, we screened for circulating anti-pituitary antibodies as well as the ectopic antigen expressions in consecutive 20 patients with ICI-related hypophysitis who showed ACTH deficiency and demonstrated that two patient sera exhibited anti-corticotroph antibodies. Interestingly, these autoantibodies recognized POMC protein, and ectopic ACTH expression was specifically detected in the tumors of these patients. These data are in line with the hypothesis that ectopic ACTH expression in tumors can evoke autoreactive T-cell activation and ICI administration can enhance the autoimmunity, ultimately resulting in the specific injury of corticotrophs and ACTH deficiency.

In CTLA-4 inhibitor-related hypophysitis, it has been reported that the CTLA-4 expression in the anterior pituitary cells evoked a direct interaction of anti-CTLA-4 antibody with these cells and induced a complement-dependent cell injury in the pituitary [5]. In contrast, the underlying mechanisms in PD-1/PD-L1 inhibitor-related hypophysitis in the present study seem to be different because PD-L1 or PD-1 is not expressed in the pituitary [24, 25]. Also, the result that only a part of patients exhibited anti-corticotroph autoantibody suggested that there are other mechanisms.

It has been reported that in patients with IAD, circulating anti-corticotroph antibody has been detected in a portion of patients [26]. In addition, an epitope of endogenous proteins has been presented by MHC class I molecules in the anterior pituitary cells [27], which enables the recognition of specific T-cell receptors on cytotoxic T cells. Interestingly, a case of acquired IAD as a form of paraneoplastic syndrome was caused by autoimmunity against corticotrophs with the ectopic ACTH expression in the complicated tumor [15], and several cases of IAD were complicated with malignant tumors have been reported [28, 29]. Interestingly, ectopic ACTH, expression is not rare in various cancers, regardless of the type and ectopic expression of the other pituitary hormones is extremely rare [15].

 These data may explain, at least in part, the reason for the preference of ACTH deficiency in ICI-related hypophysitis. We also detected anti-somatotroph antibody in one patient. ACTH, TSH, and LH/FSH deficiency, but not GH deficiency, were observed in this patient, and the ectopic expression of GH was not detected in the tumor. Although further investigation is necessary to clarify the significance of this anti-GH antibody, it is speculated that it did not play a pivotal role in the development of hypophysitis, rather, it may be results of destruction of the pituitary.

Generally, pharmacological dose of glucocorticoids is used as first-line therapy for treatment of irAEs. However, in terms of ICI-related hypophysitis, it is recommended to use physiological doses of glucocorticoids as a replacement therapy unless the presence of severe visual disturbance or intolerable headache because pharmacological dose of glucocorticoids does not restore pituitary function and recent study suggested high-dose glucocorticoids in patients with ICI-related hypophysitis might impair the effect of ICIs and survival [30, 31].

One limitation of the study is the sensitivity of autoantibody. The sensitivity for detecting the anti-pituitary antibody may not be enough because of the use of mouse pituitary tissue. The other limitation is the number of patients. However, considering the prevalence of hypophysitis with a strict diagnosis, it may be difficult to recruit more number of patients.

## Conclusion

We demonstrated that 10% of PD-1/PD-L1 inhibitors-related hypophysitis were associated with the autoimmunity against corticotrophs and maybe caused as a form of paraneoplastic syndrome, in which ectopic expression of ACTH in the tumor was observed. It is also suggested that the pathophysiology is heterogenous in ICI-related hypophysitis. Although further investigation is necessary, these data provide insight into a novel mechanism for the etiology of irAE.

## Supplementary Information

Below is the link to the electronic supplementary material.

Supplementary file1 (PDF 419 kb)

## Data Availability

All data associated with this paper can be found in the main text.

## Abbreviations

ACTH: Adrenocorticotropic hormone
APA: Anti-pituitary antibody
CLEIA: Chemiluminescent enzyme immunoassay
CLIA: Chemiluminescent immunoassay
CRH: Corticotropin-releasing hormone
CTLA-4: Cytotoxic T-lymphocyte antigen-4
ECLIA: Electrochemiluminescence immunoassay
EDTA,: Ethylenediaminetetraacetic acid
E2: Estradiol
Free T4: Free thyroxine 4
FSH: Follicle-stimulating hormone
GnRH: Gonadotropin-releasing hormone
GHRP-2: Growth hormone-releasing peptide-2
irAE: Immune-related adverse event
IAD: Isolated ACTH deficiency
ICI: Immune checkpoint inhibitor
LH: Luteinizing hormone
LHRH: Luteinizing hormone-releasing hormone
PD-1: Programmed cell death-1
PD-L1: Programmed cell death 1-ligand 1
POMC: Proopiomelanocortin
PRL: Prolactin
TSH: Thyroid-stimulating hormone
TRH: Thyroid-releasing hormone
