# Supplementary figures and images for: Mechanistic insights into immune checkpoint inhibitor-related hypophysitis: a form of paraneoplastic syndrome
Source: Cancer Immunol Immunother. 2021 May 11;70(12):3669–77. doi: 10.1007/s00262-021-02955-y (PMC8571153; doi:10.1007/s00262-021-02955-y)

**Supplementary Fig. 1**

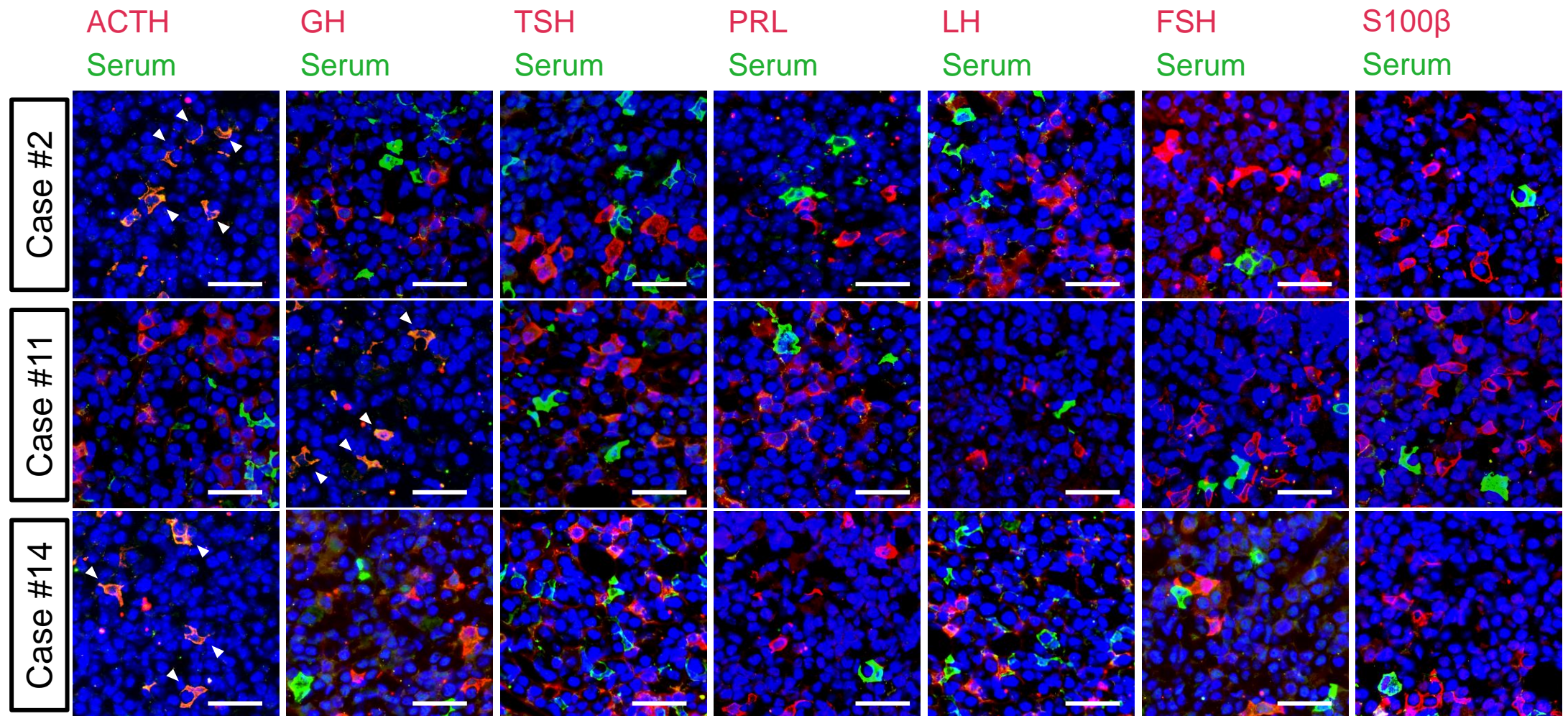

Supplement: Supplementary file 1 — Supplementary file1 (PDF 419 kb) [file 262_2021_2955_MOESM1_ESM.pdf]
